# Sensitivity to electricity – Temporal changes in Austria

**DOI:** 10.1186/1471-2458-8-310

**Published:** 2008-09-12

**Authors:** Joerg Schröttner, Norbert Leitgeb

**Affiliations:** 1 Institute of Health Care Engineering, Graz University of Technology, Graz, Austria

## Abstract

**Background:**

An increasing number of persons suffer from non-specific health symptoms such as headache, sleep disturbances, difficulties in concentrating and more. In lack of a medical explanation, more and more persons take refuge to the assumption that they were electromagnetic hypersensitive (EHS) and electromagnetic pollution causes their problems. The discussion whether electromagnetic fields (EMF) could cause such adverse health effects is still ongoing.

**Methods:**

Based on the Austrian inhabitants a statistical cross-sample of the general population with regard to age, gender and federal state had been investigated to assess the actual situation and potential temporal changes in comparison with a former study of 1994. In a telephone survey a total number of 526 persons were included.

**Results:**

This study showed an actual EHS prevalence of 3.5% compared with 2% estimated in 1994. About 70% of the sample believed that electromagnetic pollution could be a risk factor for health. More than 30% declared to at least some degree to be concerned about their well-being near mobile phone base stations or power lines. However, only 10% were actively looking for specific information. Media triggered EHS hypothesis in 24% of the cases.

**Conclusion:**

The results show that concerns about EMF did not decrease with time in spite of scientific studies and health risk assessments concluding that a causal relationship of EMF below recommended reference levels and non-specific health symptoms would be implausible.

## Background

An increasing number of persons suffer from non-specific health symptoms such as headache, sleep disturbances, difficulties in concentrating and more. In lack of a medical explanation, more and more persons take refuge to the assumption that they were EHS and electromagnetic pollution causes their problems. So far, the results of experimental studies on EHS have been contradictory [1-8], leaving it still open whether a causal link between exposure to electromagnetic fields (EMF) below the recommended reference levels and reported health symptoms actually exists.

WHO proposed to replace the term "electromagnetic hypersensitivity" (EHS) by the term "Idiopathic Environmental Intolerance with attribution to EMF" (IEI-EMF), in order to avoid the term EHS, which implies that a causal relationship between reported symptoms and EMF has already been established [9]. WHO concluded, that IEI-EMF cases are not able to detect EMF exposure more reliably than other individuals, and symptoms do not seem to be correlated with EMF exposure. However, there are people suffering from unspecific health symptoms and claiming to be electromagnetic hypersensitive. To assess the prevalence of EMF-related problems an inquiry among Austrian general practitioners has been recently performed [10]. The results showed that there is a widespread contradiction between physician's opinion and the judgement of national and international health risk assessment bodies. An overwhelming percentage of general practitioners (96%) to at least some degree believed in the effects of environmental electromagnetic fields on health, and only 39% have never associated health symptoms with electromagnetic pollution. A similar discrepancy between physician's opinions and established scientific assessment was shown in an inquiry study including 342 interviews of physicians in Switzerland [11].

In 1994 Leitgeb [12,13] investigated the EHS issue and first estimated the potential EHS subgroup to be less than 2% of the general population. This conclusion followed from two separate investigations. In the first, 200 persons, which were randomly selected among the rural and urban clients of a power utility, were investigated [12]. Their individual sensitivity was assessed by self classification. This study resulted in 10% of people classifying themselves as "very sensitive" and about 14% of people classifying themselves as "sensitive" to EMF. In lack of EMF-perception their self-definition was derived from other not EMF-related clues like general sensitivity or existing allergies. Based on comparisons with measured electrosensitivity it could be concluded, that the estimated percentage of self-declared EHS is unreliable and usually considerably overestimated. Electrosensitivity (the ability to perceive electricity) was assessed by a quantitative parameter namely the perception threshold of directly applied electric 50 Hz currents [13]. The analysis of double-blind perception measurements at a sample of 606 persons indicated the presence of a subgroup of 2% with significantly increased sensitivity. Further investigations [14,15] of electric current perception thresholds on an extended sample of the general population confirmed the initial finding of a 2% electro-sensitive subgroup with significantly increased perception threshold. It was concluded that the potential size of the EHS prevalence should be less than 2% of the general population, since significantly increased electro-sensitivity was assumed to be a necessary, but not sufficient precondition for EHS [14].

Other population-based surveys have been performed in Sweden, California and Switzerland. In 1997 Hillert et al. [16] conducted a cross sectional questionnaire study among 15000 adults in Stockholm County. The response rate was 73%. Of the respondents 1.5 percent reported hypersensitivity to electric or magnetic fields. Levallois et al. [17] reported results of a telephone survey among a sample of 2072 Californians. This survey found an EHS prevalence of 3.2 percent (68 persons) who reported being allergic or very sensitive to electrical devices. In 2004 a telephone interview of a representative sample of the Swiss population was conducted by Schreier et al. [18] who reported a prevalence of 5% EHS in Switzerland. In Germany 51444 persons from 14 to 69 years were included in a cross sectional study in 2006 [19]. The response rate was 58.4% representing 30047 interviews. Of the respondents 16.9% reported concerns about EMF from mobile phone base stations and 9.5% reported on impaired health attributed to EMF from transmitting stations.

The aim of the present survey was to get actual data and to assess potential temporal changes of the prevalence of EMF-related concerns and hypersensitivity compared to the initial study of 1994.

## Methods

A telephone survey on a cross-sectional sample of the Austrian population was performed. In order to generate a representative sample, a computer controlled random generator algorithm was used to identify individuals from the public telephone registry and selection was made to represent Austrian's inhabitants with regard to age, gender and federal province. Therefore, based on regional data of the federal provinces [20] the age distribution of the whole Austrian population in five year age classes from 15 to 80 years was determined. With that result an adjusted sample of 526 persons was generated, which was interviewed in a telephone survey. Telephone numbers were randomly selected from the public telephone registry. Once a household was reached, the person answering the phone call was asked for his/her age. If the person fitted into the sample the interview was performed. If the person did not fit to the sample or his/her age class was already filled, the person was asked for another cohabitant older than 14 years, who is available for an interview. If none of them fitted to the sample, another call was made. In that manner the predetermined regional composition (federal state) and size of age classes were randomly and completely filled.

**Figure 1:**
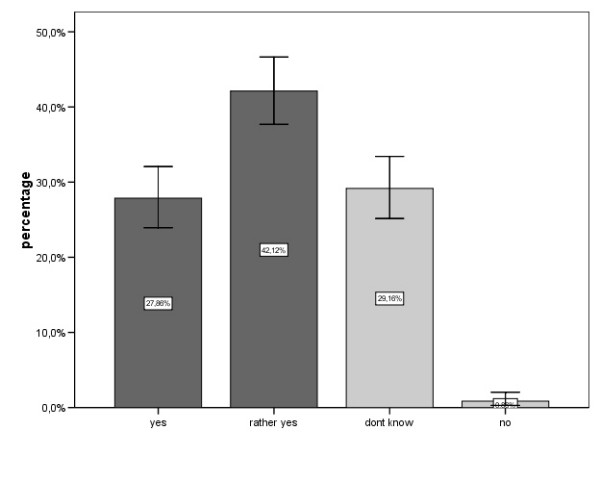
Answers to the question: „Do you think electromagnetic pollution could be a risk factor for health?“.

**Figure 2:**
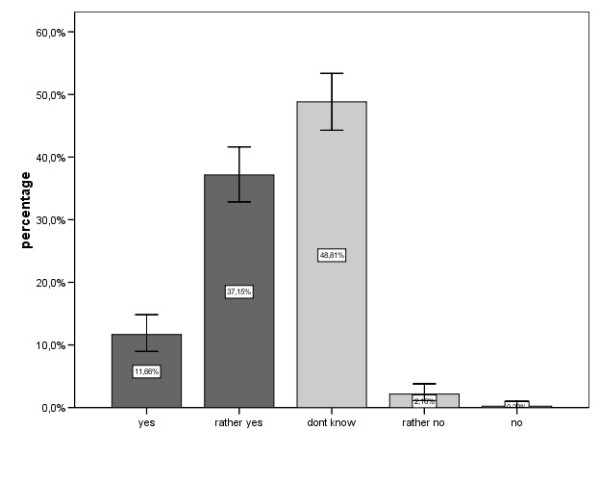
Answers to the question: „Do you think electromagnetic pollution could enforce symptoms of diseases and allergies?“.

**Figure 3:**
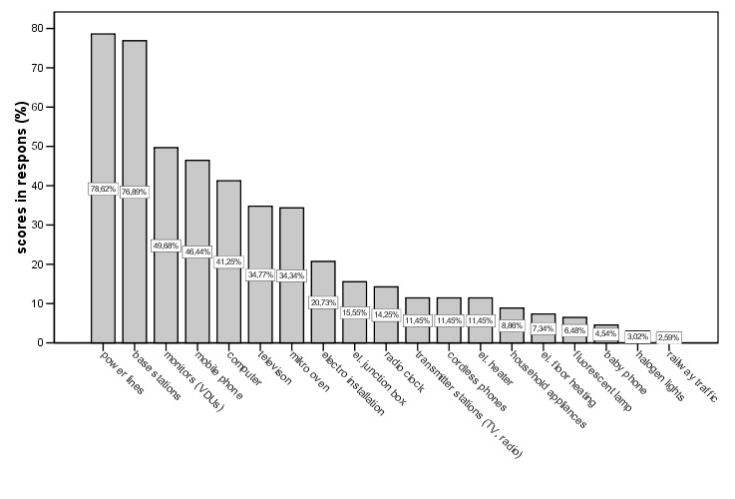
Response to the question: „Which sources do you consider as responsible for electromagnetic pollution?“.

A two-page questionnaire was developed comprising 25 questions [see Additional file 1]. The first part of the questionnaire concentrated on health status and lifestyle. Questions like "How would you rate your health status during the last month?" were asked. For this example predetermined choices were given with "very bad", "bad", "satisfying", "good", or "very good". The second part comprised EMF risk perception and avoidance behaviour, the perception of critical EMF sources and potential EMF related symptoms. Questions like "Do you think electromagnetic pollution could be a risk factor for health?“, „Do you think electromagnetic pollution could enforce symptoms of diseases and allergies? or „Which sources do you consider as responsible for electromagnetic pollution?” were asked (see figure 1 to 3). Persons were classified as EHS if they reported adverse health effects from EMF sources ("Do you feel disturbed from electromagnetic pollution?, If yes, which symptoms do you relate to electromagnetic pollution?") and suffered to such a high degree, that they were actively looking for medical help. Reported symptoms were asked in an open way. Finally, general questions were included such as education, employment status and living conditions. The study was performed in compliance with internationally recognized guidelines. The design was approved by the Ethic Commission of the Medical University Graz (Reference number: 19–277).

Data were analysed using the statistical software SPSS (SPSS 12.0, SPSS Inc. Headquarters, Chicago, Illinois 60606). Non-parametric Chi-square test was used to identify significant differences of groups within the investigated sample. Estimated prevalence values are presented with 95% confidence intervals (CI 95%), and p < 0.05 is considered as statistically significant.

## Results

All together 526 interviews were carried out. 460 persons (88%) answered all questions and in 66 cases (12%) some of the answers were refused. The interview took about 20 minutes per person without EMF problems, and about 10 minutes more for hypersensitive persons.

### General data and health status

The most frequent health complaints were *meteorosensitivity *(16.4%) followed by *migraine *(14%), *sleep disturbances *(8.2%) and *headache *(8%). Allergic reactions were listed by 43%, primarily to *pollen *(13.7%), *earth rays *(10.3%), *food *(9.1%) and *pharmaceuticals *(7%).

Concerning life style, 85% of the persons were healthy eaters. However, about a quarter never participated in sports, more than 85% drank alcohol occasionally and about 50% of the sample were smokers.

The answers concerning personal use of electrical equipment showed that 95% used radio and television sets, 79% a mobile phone, 57% a personal computer and 54% cordless telephones. At their workplaces 56% used mobile phones and computers.

### Risk perception

70% of the study population believed to some degree that electromagnetic pollution is a health risk factor, while only less than 1% denied it (Fig. 1).

Slightly less than 49% agreed that electromagnetic pollution could worsen existing health symptoms, while 48.8% did not know and only 2.3% denied (Fig. 2).

It is remarkable that more than 30% (95% CI: 26.1–34.4) declared to some degree (15.1%) or completely (15.1%) that they felt uncomfortable near mobile phone base stations or power lines. Actually 13.6% (95% CI: 10.5–16.7) claimed to feel nearby electrical equipment. 29% (95% CI: 25.2–33.5) were not convinced that adverse health effects of electromagnetic pollution are negligible compared to other environmental influences, while 24% (95% CI: 19.7–27.4) were convinced and 47% (95% CI: 42.5–51.6) had no opinion.

Nearly every fourth (24%, 95% CI: 20.3–28.1) agreed to some degree, that he/she would accept a higher health risk of new technologies for increased comfort they provided.

The dominant information sources were public media for 24% (95% CI: 19.5–27.2), while only 10.4% (95% CI: 7.6–13.1) were actively looking for information by their own.

The most frequently identified health-relevant EMF sources were power lines (79%), mobile phone base stations (77%), video display units (50%), mobile phones (46%) and computers (41%). (Fig. 3)

Electrical equipment is avoided by 28% (95% CI: 27.3–35.8), mainly by restricted use and turning the appliances on and off (each 32%), followed by de-plugging the equipment (26%) and installing a circuit control switch (22%).

### EHS Prevalence

In this survey 2.1% (95% CI: 1.0–3.8) of the investigated sample felt very intensely disturbed by EMF and another 27.2% (95% CI: 26.7–35.1) argued to be slightly disturbed by electromagnetic pollution. From this 29.3%, 16 persons, representing 3.5% (95% CI: 1.9–5.1) of the study group, claimed to be electromagnetic hypersensitive to such a high degree that they asked a physician for help for their EMF-associated health problems. Most frequently mentioned symptoms with regard to EHS were sleep disturbances, migraine, nervousness and tinnitus.

EHS was most prevalent among middle-aged persons and persons with a higher education. The age group with the highest prevalence of 6.3% (95% CI: 2.5–10.1) was between 31 and 45 years. The results showed that education levels seem to influence EHS prevalence. Whereas only 1% (95% CI: 0.0–2.4) of the responders who did an apprenticeship were EHS persons, academics, which completed a degree at a university or a university of applied science, showed a 12 times higher percentage. The data showed that prevalence of hypersensitivity seems to be higher among people living in the city or at countryside than those living in the suburbs (Table 1).

**Table 1:** Prevalence's of self reported hypersensitivity of various demographic groups

	group	number	EHS in % (number)	95% CI (%)
	all	526	3.5 (16)	1.9–5.1

gender	male	259	3.6 (8)	1.3–5.9
	female	267	3.3 (8)	1.2–5.4

age	15 to 30 years	123	1.0 (1)	0.0–2.8
	31 to 45 years	161	6.3 (9)	2.5–10.1
	46–60 years	119	3.4 (4)	0.1–6.7
	> 61 years	123	1.9 (2)	0.0–4.3

education	comprehensive school	60	5.0 (3)	0.0–10.5
	apprendiceship	206	1.0 (2)	0.0–2.4
	high school	147	3.4 (5)	0.5–6.3
	university	50	12.0 (6)	3.0–21.0
	*missing*	*63*	-	-

place of residence	city	163	3.7 (6)	0.8–6.6
	suburb	178	2.8 (5)	0.4–5.2
	countryside	122	4.1 (5)	0.6–7.6
	*missing*	*63*	-	-

(Numbers of persons are given in brackets).

Statistical analyses with nonparametric Chi-square test found significant differences (p < 0.001) in regard to different education levels and age groups. In both cases the pre-conditions for Chi-square testing were not fulfilled because of low cell frequencies. Therefore Chi-square test with pooling was used. In case of education levels the 'comprehensive school' group and the 'apprenticeship' group were pooled, as well as the 'high school' group and the 'university' group. For age two groups were generated, whereas one group includes persons from 15 to 45 years and a second group people over 45 years. After pooling both results remained significant, with p = 0.006 for education level and p = 0.004 for age. No statistically significant difference (p = 0.117) was found between groups regarding the place of residence.

It has to be taken into account that about 40% of the persons aged between 31 and 45 were living in the city, many of them with higher education. A correlation analyses confirmed the relation between the parameters place of residence, education and age with a statistically significant result at a significance level of p = 0.01.

## Discussion

This study shows that the actual percentage of persons claiming to be electromagnetic hypersensitive amounts to 3.5%, which is almost twice compared to the potential of 2% formerly estimated by Leitgeb in 1994, based on measurements of the electric current perception [12,13]. A comparison of reported EHS prevalence's of different studies faces methodological problems, because objective EHS-criteria are lacking and classifications as EHS are quite different among the studies. For example, Levallois et al. [17] defined EHS persons as (citation) "allergic or very sensitive to getting near electrical appliances, computers or power lines" when reporting a prevalence of 3.2%. In the present study a similar question ("Do you feel disturbed in your well-being near mobile phone base stations or power lines?") was asked and resulted in 15% feeling disturbed in their well-being near mobile phone base station or power lines. Unfortunately, yet there is no commonly accepted definition of EHS leading to a span of reported prevalence from 1.5 to 9.5% [16-19]. The criterion of EHS in this study was that persons had to suffer to such a high degree, that they were actively looking for medical help. Therefore, it is not surprising that the results of this study are at the lower end of the span.

Care was taken to generate a representative sample. The stratified sampling approach has been preferred over contacting a preselected sample to overcome the more relevant bias of poor response rates. A potential bias due to persons staying more or less time at home could not be avoided by either approach. The chosen approach was to randomly and completely fill predetermined sample subgroups which were adjusted for age, gender and region. Therefore, phone calls were made until the representative sample was completed. The participation rate was 35%. About 1500 phone calls were necessary to complete the sample. However, some bias might still be left. First, individuals having no telephone were not included in this study. Since other studies demonstrated that, if there is a difference at all, EHS prevalence is expected to be higher within low income groups [16,17]. Therefore, if at all, this potential bias could cause under representing concerned people. Second, concerned persons might have been more willing to accept the interview hence leading to overestimating concerns. Third, mobile phones might be avoided by people assuming adverse EMF health effects, therefore including mobile phone users might lead to bias. The used telephone registry contained about 4.7 billion numbers (2.3 billion fixed-line phones and 2.4 billion mobile phones). Apart from the fact that concerns do not necessarily motivate not to have any mobile phone but might lead to restrictive use. Therefore, if at all, mobile phone-based interviews could have led to underestimating concerns. This potential bias would not challenge the reported increase.

**Figure 4:**
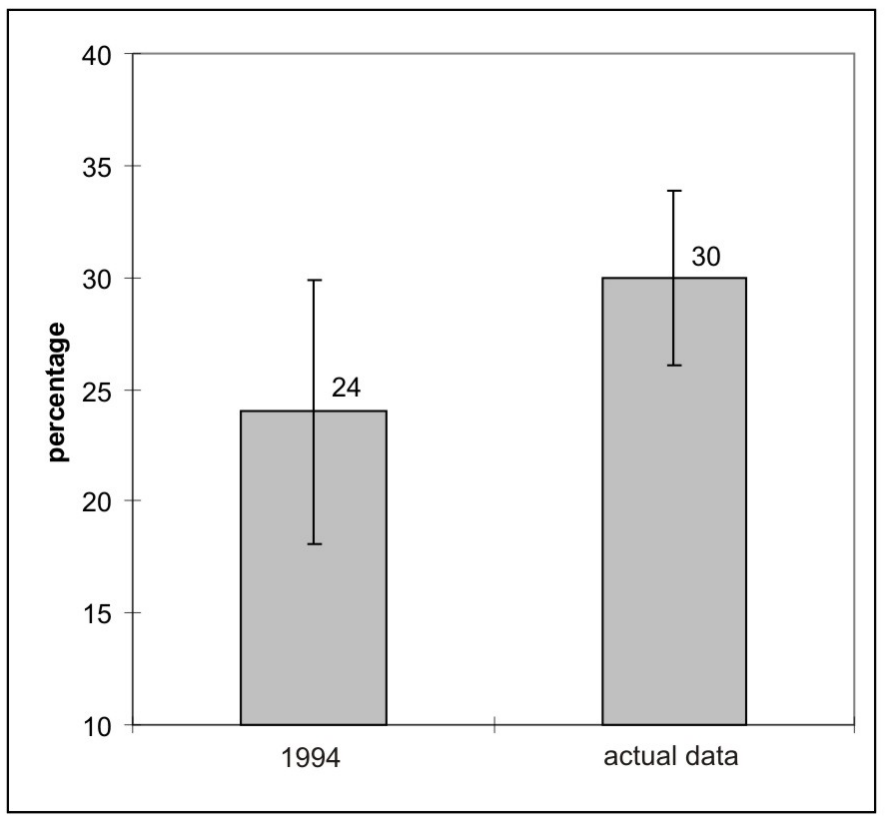
Percentage (and 95% confidence interval) of self-reported sensitivity to EMF compared between the actual data and the results from 1994.

2.1% of the investigated sample felt very intensely disturbed by EMF (2.6% women and 1.5% men). The higher percentage of women reporting EHS to electromagnetic fields is in agreement with the literature [14,16-18,21-24]. The assessment of self-classified sensitivity to EMF showed that about 30% claimed to feel the presence of electrical equipments or declared that their well-being would be impaired near mobile phone base stations and/or power lines. This is a 6% increase compared to the study of 1994 where 24% declared to be sensitive to electricity [12]. In spite of the different confidence intervals due to the different sample size of either study this constitutes a increase over time (Fig. 4).

EHS most frequently reported symptoms such as sleep disturbances, migraine, nervousness and tinnitus. The dominant attributed sources were micro oven, power lines, TV and video display units, mobile phone base stations or mobile phones. These results are in consistence with results of the literature. Sleep disorders and headache as primary symptoms were also found by Schreier et al. [18] attributed to power lines and mobile phone headsets. Hillert et al. [16] found that the most commonly reported symptoms were fatigue, facial skin problems or eye irritations, while headache ranked on place six. This difference can be explained, because in Sweden was work with a video display unit (VDU) the most frequently reported triggering factor for EHS [21] with the typical symptom cluster including skin problems and eye irritations.

The results also showed that in the group of persons between 31 and 45 years of age the percentage of EHS raises up to 6.3%, and even up to 12% in the group of academics. Statistical analyses could identify significant differences in regard to the different education levels and age, but found no significant difference in regard to place of residence (urban and rural areas). The highest EHS prevalence was found in the age class 31 to 45 years. These findings are consistent with results from Schreier et al. [18], who reported, that both higher-educated persons and persons above the age of 35 represent a large percentage of the EHS group. A different trend was reported by Levallois et al. [17] and Hillert et al. [16]: The age groups over 60 years exhibited the highest EHS prevalence. In the study from Levallois et al. [17] a statistical association of the EHS prevalence with low socio-economic factors and ethnicity was reported. The authors mentioned being unable to work, being from a race/ethnicity and having low income as EHS co-factors. Hillert et al. [16] also found an association of EHS with low income. Ethnicity as a potential EHS co-factor like in California was no issue in Austria, because of insignificant other ethnicities. It needs to be acknowledged that there are structural differences of the population between California and Austria.

In addition the study showed that a considerably high percentage of the whole study population is convinced that electromagnetic pollution could be a health risk factor. This is remarkable since until now below recommended reference levels no causal relationship between electromagnetic fields and adverse health effects has been proven, nor can it be explained by existing interaction mechanisms [9,25].

## Conclusion

The objective of the study was to provide updated information and to investigate potential temporal change of the prevalence of EMF-related concerns and hypersensitive persons in Austria since a first study in 1994. It could be shown that the percentage of persons convinced to be sensitive to electricity has increased. In addition, it could be shown that 3.5% persons suffer from adverse health effects attributed to EMF. This is almost twice as the prevalence of 2% estimated in 1994. The results show that concerns about EMF did not decrease with time in spite of scientific studies and health risk assessments concluding that a causal relationship of EMF below recommended reference levels would be implausible.

## Competing interests

The authors declare that they have no competing interests.

## Authors' contributions

JS participated in the design of the study, the development of the questionnaire, performed the statistical analysis and drafted the manuscript. NL conceived and supervised the study, participated in the design, the development of the questionnaire and writing the paper. All authors read and approved the final manuscript.

## Pre-publication history

The pre-publication history for this paper can be accessed here:



## Supplementary Material

Additional file 1 questionnaire. This file contains the questionnaire which was used in this study. Click here for file
