# Supplementary material for: Sensitivity to electricity – Temporal changes in Austria
Source: BMC Public Health. 2008 Sep 12;8:310. doi: 10.1186/1471-2458-8-310 (PMC2562386; doi:10.1186/1471-2458-8-310)
Supplement: Additional file 1 — questionnaire. This file contains the questionnaire which was used in this study. [file 1471-2458-8-310-S1.pdf]

1. **Wie bewerten Sie ihren Gesundheitszustand während des letzten Monats?**

Sehr schlecht	Schlecht	Zufriedenstellend	Gut	Sehr gut
☐	☐	☐	☐	☐

2. **Haben sie regelmäßig auftretende gesundheitliche Beschwerden unklarer Ursache?**

☐ Migräne ☐ Wetterfühligkeit ☐ Schlafstörungen ☐ Kopfschmerzen
☐ Andere: ____

3. **Reagieren sie allergisch/überempfindlich auf**
(Mehrfachnennungen möglich)
☐ Tiere/Tierhaare ☐ Pollen/Gräser ☐ Staub ☐ Schimmel
☐ Medikamente ☐ Nahrungsmittel ☐ Wasseradern ☐ Kosmetika
☐ Nickel ☐ Amalgam (Zahnfüllung)
☐ Anderes: ____

4. **Was sind Ihre Lebensgewohnheiten?**

	Nie	Gelegentlich	Häufig
Ernähren Sie sich gesund (biologisch)?	☐	☐	☐
Treiben Sie Sport?	☐	☐	☐

	Nie	Gelegentlich	Regelmäßig
Rauchen Sie ?	☐	☐	☐
Trinken Sie Alkohol ?	☐	☐	☐

5. **Benutzen Sie folgende elektrische Geräte ?**
(Mehrfachnennungen möglich)

	Am Arbeitsplatz	Im Wohnbereich
PC	☐	☐
Überwachungsmonitor/Fernseher	☐	☐
Mobiltelefon (Handy)	☐	☐
Schnurlostelefon	☐	☐
Elektroheizung, el. Heizstrahler	☐	☐
Mikrowellenherd	☐	☐
Radiowecker	☐	☐

6. **Bitte bewerten Sie folgende Aussagen:**

	Stimmt völlig	Stimmt teilweise	Weiß nicht	Eher nicht	Stimmt nicht
Elektrosmog kann ein Risiko für die Gesundheit darstellen.	☐	☐	☐	☐	☐
Ich spüre, wenn in meiner Nähe ein Elektrogerät benützt wird.	☐	☐	☐	☐	☐
Elektrosmog könnte die Symptome von Krankheiten oder Allergien verstärken.	☐	☐	☐	☐	☐
Für die Annehmlichkeiten der modernen Technik würde ich ein höheres Gesundheitsrisiko in Kauf nehmen.	☐	☐	☐	☐	☐
Die Gefährdung der Gesundheit durch Elektrosmog ist im Vergleich zu anderen Umwelteinflüssen zu vernachlässigen.	☐	☐	☐	☐	☐
In der Nähe von Hochspannungsleitungen oder Handymasten fühle ich in meinem Wohlbefinden gestört.	☐	☐	☐	☐	☐
Berichte in den Medien brachten mich darauf, dass gesundheitliche Probleme Elektrosmog als Ursache haben könnten.	☐	☐	☐	☐	☐
Ich habe schon aktiv nach Informationen zum Thema Elektrosmog gesucht.	☐	☐	☐	☐	☐
Ich habe Schlafprobleme, wenn in meiner Nähe elektrische Geräte eingeschaltet sind.	☐	☐	☐	☐	☐
Ich habe bereits einen Arzt zum Thema Elektrosmog konsultiert.	☐	☐	☐	☐	☐

7. Welche elektrischen Geräte sehen Sie als Hauptverursacher von Elektrosmog an?

☐ Babyfon ☐ Elektroinstallation ☐ Halogenlampen ☐ Radiowecker
☐ Bahnverkehr ☐ Elektroverteiler ☐ Haushaltsgeräte ☐ Rundfunksender
☐ Computer ☐ Fernseher ☐ Hochspannungsleitungen ☐ Schnurlostelefon
☐ el.Fußbodenheizung ☐ Handy ☐ Mikrowellenherd ☐ Leuchtstofflampen
☐ el. Heizstrahler ☐ Handymasten ☐ Monitore
☐ Sonstige: ____

8. Kennen Sie Personen, die sich durch Elektrosmog gestört fühlen?

☐ Ja ☐ Nein Falls ja, wie viele Personen in ihrem Bekanntenkreis sind davon betroffen? ____ Personen

9. Fühlen Sie sich durch Elektrosmog gestört?

Gar nicht	Etwas	Stark
☐	☐	☐

Falls ja, welche Ihrer Symptome bringen Sie mit Elektrosmog in Zusammenhang?
(bitte nutzen Sie die vorgesehenen Zeilen)

____________________ # ____________________ # ____________________

____________________ # ____________________ # ____________________

10. Tun Sie etwas um Elektrosmog zu verringern, und falls ja, was?
(Mehrfachantworten möglich)

☐ Benutzung von Elektrogeräten vermeiden ☐ Entstörgeräte verwenden
☐ Netzfreischalter installieren ☐ Geräte nicht mit der Fernbedienung abschalten
☐ Unbenützte Geräte abstecken
☐ Etwas anderes, und zwar: ____

11. Falls Sie Beschwerden haben: Wie haben sie entdeckt, dass diese mit Elektrosmog zusammenhängen?

☐ Eigene Beobachtung ☐ Hörensagen ☐ Radio, Fernsehen ☐ Zeitungen, Zeitschriften
☐ Produktwerbung ☐ Arzt ☐ Anders: ______________________

12. Persönliche Daten

Alter: ___ Jahre
Geschlecht ☐ m ☐ w
Größe: ___ cm
Gewicht: ___ kg

13. Höchste abgeschlossene Ausbildung?

☐ Pflichtschule ☐ Lehre ☐ Matura ☐ Hochschule

14. Welcher Berufsgruppe gehören Sie an?

☐ Arbeitslos ☐ Arbeiter ☐ Hausfrau/mann ☐ Pensionist
☐ in Ausbildung ☐ Angestellter ☐ Selbstständig

15. Arbeits- bzw. Wohnort ?
(Anleitung: bitte nach Ihrer Einschätzung ein Kreuz setzen)

Sie wohnen
am Land in der Stadt

Sie arbeiten
am Land in der Stadt

Wir danken für ihre Mithilfe!
